# Locomotion behavior of dairy cows on traditional summer mountain farms in comparison with modern cubicle housing without access to pasture

**DOI:** 10.1371/journal.pone.0264320

**Published:** 2022-03-09

**Authors:** Maher Alsaaod, Salome Dürr, Damian Iten, Wolfgang Buescher, Adrian Steiner

**Affiliations:** 1 Clinic for Ruminants, Vetsuisse-Faculty, University of Bern, Bern, Switzerland; 2 Veterinary Public Health Institute, Vetsuisse-Faculty, University of Bern, Bern, Switzerland; 3 Livestock Technology Section, Institute for Agricultural Engineering, University of Bonn, Bonn, Germany; Michigan State University, UNITED STATES

## Abstract

Pasture based systems enable cattle to express their natural behavior and are thus expected to provide better welfare than the majority of confinement systems. The aim of this study was to objectively measure locomotion activity of healthy dairy cattle kept on mountain pastures (n = 44) compared with cows kept in cubicle housing systems (n = 38). Selected cows were equipped with a validated 3D-accelerometer on one hind limb, and locomotion behavior was recorded for 48 hours. The 1-hour summaries of the variables lying time, walking time, standing bouts, walking bouts and number of strides were summed up to 24-hour summaries, and the means of the stride distance and stride duration were weighted by the variable number of strides. Mountain pasture cows had higher locomotor activity levels in comparison to cubicle cows. Mountain pasture cows spent less time lying down (528.1±109.5 min/day vs. 693.3±73.8 min/day; *P*<0.0001) and more time walking (75.6±25.9 min/day vs. 38.8±15.8 min/day; *P* <0.0001) than cubicle cows. Lying bout duration was longer in cubicle than in mountain pasture cows (90.9± 15.2 min/bout vs. 74.2 ± 21.1 min/bout; *P* = 0.0001), whilst the number of walking bouts was higher in mountain pasture cows than cubicle cows (199.1 ± 49.1 vs. 123.8 ± 43.8 bouts per day; *P* < 0.001). Likewise, the number of strides was higher in mountain pasture cows than cubicle cows (2040.5 ± 825.3 vs. 916.7 ± 408.6; P < 0.001). Mountain pasture cows had shorter stride duration (*P* < 0.0001) and shorter strides (*P* = 0.0002) than cubicle cows (1.8 ± 0.1 s/stride vs 2 ± 0.2 s/stride and 126.3 ± 18.1 vs 142.1 ± 17.8 m/stride, respectively). In summary, cows kept on mountain pasture were more active and spent longer than 12 hours / day standing. Lying markedly less than 12 hours per day seems to represent the normal behavior of pastured cows searching for fresh grass. This does not cause any obvious damage to the locomotor system as claws of cattle are well adapted to long periods of movement on mountain pastures.

## Introduction

Welfare of intensively farmed animals and whether the housing systems allow individuals to express natural behavior, are of public concern [1, 2]. Pasture based husbandry systems enable cattle to express their natural behavior such as grazing, freedom of movement and social interaction and thus provide better welfare than confinement systems [3–5]. However, due to the increasing use of conventional indoor housing systems and automated milking systems combined with continuously increasing herd size, zero grazing systems are becoming more and more common in many countries of the European Union (EU). There is a clear trend of a reduction of the proportion of grazing versus indoor feeding time [6]. According to Reijs et al. [7], proportions of dairy cows with access to pasture reduced from 52% in 2008 to 35% in 2012. Until 2025, the number of dairy cows in the Netherlands with access to pasture are expected to be reduced by half within ten years [8].

Furthermore, over 75–80% of all lactating dairy cows in Canada and United States are housed in zero-grazing systems [9, 10], while access to pasture ranges from 15 to 30% in Germany, 68% in Austria to 80% in Switzerland of total dairy cattle housed [11].

Many modern housing systems involve keeping animals indoors and since the 1970s, cubicle housing systems (or "freestall barns" or "cubicle houses") have been widespread in many countries [12]. Housing dairy cows indoor allows for the control of climatic and environmental factors including adjusted feed supply, temperature, humidity, bedding availability, and regular health checks [13]. However, the welfare of indoor housed cattle may be compromised as the ability of the animals to express their normal behavior is restricted and is often accompanied by an increased prevalence of various diseases [14–17]. For example, a study by Olmos et al. [18] showed that cubicle housed cows were more likely to be clinically lame in comparison to permanent pastured cows (prevalence 61% vs. 17%, respectively). A recent study revealed that regular access to pasture has positive effects on welfare indicators in dairy cows, while those effects are not observed when the cows are kept indoors [19]. Alternatively, free-walk housing systems such as “bedded pack barns” or “compost/composting barns” are promising animal-friendly housing systems that can improve animal welfare by providing more space per animal, soft bedding, and unrestricted roaming [20].

In comparison to conventional indoor housing systems, allowing cows access to pasture offers comfortable lying [14] and locomotion options for dairy cows [21]. However, data regarding the activity "time-budget" of dairy cows kept on traditional summer pastures is not available. Mountain summer pasturing is an ancient traditional management system, where cows are kept outdoors in high altitude (1000 to 2000 meters over sea level) pastures during the daytime and kept inside in tie stalls at night between the evening and morning milkings. The objective measurement of locomotion activity of dairy cows has to be highly accurate, reliable and specific for an extended set of behavioral variables. The new generation of the RumiWatch^®^ three-dimensional (3D)-accelerometers fulfill these criteria [22, 23].

The aims of the current study were to (i) objectively measure the locomotion activity of healthy dairy cows kept on traditional mountain summer pastures, using validated accelerometers; (ii) establish a basic measurement of locomotion behavior of dairy cows kept in a traditional housing system (iii) and to compare the characteristics of locomotion behavior on mountain pasture to those of cows kept on typical modern cubicle housing systems. The data collected will increase our knowledge and enable us to objectively evaluate differences in bovine behavior within both ancient and modern housing systems.

## Materials and methods

### Ethical statement

The study protocol was approved by the animal experimentation committees of the cantons of Berne, Switzerland (permission # BE38/18) and Zurich (Switzerland; ZH061/15, approval no. 26475).

### Farms and animals

Data were collected from cows in two different housing systems. The farms in both housing systems were selected by convenience sampling. Verbal informed consent was obtained from the animal owners for the use of their animals in this study.

For mountain pasture, the study was carried out in July 2018 and in August 2019 on five dairy farms from two alpine pastures in Switzerland. The pasture was located in mountain zones around 1600 meters above sea level. Cows were milked twice per day at 06:00 AM and 7:00 PM. After the morning milking, they were let outside one after the other to the pasture to roam freely. At around 7 PM, the cows returned to the barns, were milked for the second time and kept tied during the night without feeding until the next milking in the morning. The cows were housed in tie-stalls from autumn to spring in farms located in lower altitudes with regular pasture access.

The study for the cubicle housing systems was carried out between October 2015 and March 2016 on eight dairy farms with indoor area with cubicle systems in Switzerland. Six farms had cubicles with deep bedding and two farms with rubber mats and thin bedding; all farms provided ≥1 cubicle per cow and permanent access to outdoor areas. Cows were milked in a milking parlor twice daily at 05:30 AM and 04:00 PM. The cows were provided ad libitum access to roughage and a concentrate feed was offered in automatic feeders. In both mountain pasture and cubicle housing systems, cows have permanent free access to water. Farm management remained unchanged during the study period; however, no access to pasture was permitted to the cows from at least 3 d before and during data collection.

The full description of the study population can be found in S1 Dataset.

### Experimental design

In both housing systems, cows were included in the study if they had no apparent disease or veterinary treatment during four weeks before data collection. All included cows were more than 12 days in milk (**DIM**) at the beginning of data collection. Additionally, all selected cows must have received corrective hoof trimming at least two weeks before data collection to avoid influencing locomotion. Each farm was visited once for data collection. Cows fulfilling the inclusion criteria were randomly selected within each farm.

### Data handling and measurement of locomotion variables

Selected cows were equipped with a 3D-accelerometer (RumiWatch^®^, ITIN+HOCH GmbH, Fütterungstechnik, Liestal, Switzerland) on a hind limb, attached proximal to the fetlock joint as described by [22] and familiarized with the sensor for at least 6 hours before the data collection. The recording period lasted for 48 hours and a mean value of two days was calculated for each parameter. After the recording period was completed, the RumiWatch^®^ pedometer was removed, and raw data were transferred via USB cable to a personal computer using a specialized software (RumiWatch Manager 2, Version 2.1.0.0, ITIN + HOCH GmbH, Liestal, Switzerland, http://www.rumiwatch.ch/). Raw data were then converted into 1-hour-summaries using the valid converter developed by Alsaaod et al. (2015) for 3D-accelerometers.

The 1-hour summaries of the variables lying time, standing time, walking time, number of standing bouts, walking bouts and strides were summed up to 24-hour summaries (Table 1). The means of the stride distance and stride duration were weighted by the variable number of strides using the following formulas as described by Beer et al. [34]:

$$Stride\;duration\;(s)=\frac{\sum_{n=1}^{24}\left({STRIDEDURATION}_{n}\times {STRIDES}_{n}\right)}{\sum_{n=1}^{24}\left({STRIDES}_{n}\right)}$$

$$Stride\;distance\;(m)=\frac{\sum_{n=1}^{24}\left({STRIDEDISTANCE}_{n}\times {STRIDES}_{n}\right)}{\sum_{n=1}^{24}\left({STRIDES}_{n}\right)}$$

**Table 1:** Variables of RumiWatch® 3D-accelerometers (RumiWatch, ITIN+HOCH GmbH, Fütterungstechnik, Liestal, Switzerland) as described and validated by Alsaaod et al. [22].

Accelerometer Variable	Definition
Lying time	Lying time per day in minutes
Standing time	Standing time per day in minutes
Walking time	Walking time per day in minutes
Lying bouts	Number of lying periods
Walking bouts	Number walking periods with at least 3 consecutive strides
Lying bout duration	Mean lying bout duration in minutes
Strides	Number of strides within walking bouts
Stride frequency	Number of strides during a walking bout
Stride duration	Mean stride duration in s
Stride distance	Mean stride distance in m

### Data analysis and statistics

Data analysis was performed in R version 4.1.0 (https://cran.r-project.org/), using the packages dplyr (https://github.com/tidyverse/dplyr), lme4 (https://github.com/lme4/lme4/), lmPerm (https://github.com/mtorchiano/lmPerm), ggplot2 (https://github.com/tidyverse/ggplot2), and stringr (https://github.com/tidyverse/stringr). Summary statistics of the RumiWatch^®^ variables (Table 1) were calculated and compared between cubicle housed group and mountain pasture group using Student’s T-test (normally distributed variables with equal variance), Aspin Welch test (normally distributed variables with unequal variance between the groups) or Wilcoxon Rank Sum test (non-normally distributed variables). This was performed for the full 24 hour data and for daytime data only (between morning and evening milkings). The latter were extracted from the full dataset by filtering data collected from 06:00 AM and 07:00 PM (mountain pasture group) and 05:30 AM to 04:00 PM (cubicle housed group), respectively, to account for the different milking times.

To adjust for the differences of DIM and lactation group (lactation group 1 = first lactation, 2 = second and third lactation, 3 = > third lactation), multivariable linear mixed effect regression model was conducted with study group (mountain versus cubicle), DIM and lactation group included as fixed effects (explanatory variables) and the farm as a random effect to account for the study design. Outcome variables were log transformed when needed (non-normally distributed residuals). For each outcome variable, models of all possible combinations of the three explanatory variables were conducted and the model with the lowest Akaike Information Criteria was selected as the final model. Breed was not tested because of collinearity with the study group.

## Results

### Study population

A total of 82 dairy cows were included in this study. Cows on the mountain pasture (n = 38) were aged from 2.6 to 12.6 years (mean ± SD: 6.5 ± 3.1), were between their first and tenth lactation (mean ± SD: 4.1± 2.9), ranged from 12 to 548 DIM (mean ± SD: 248.1± 91.2), and belonged to the breeds Swiss Fleckvieh (n = 20), Simmental (n = 15), and Red Holstein (n = 3). Cows kept in cubicle housing (n = 44) were aged from 2.6 to 13 years (mean ± SD: 5.8 ± 2.6), were between their first and tenth lactation (mean ± SD: 3.5 ± 2.2), ranged from 67 to 294 DIM (mean ± SD: 172.8 ± 58.5), and belonged to the breeds Brown-Swiss (n = 20), Fleckvieh (n = 1), Holstein-Friesian (n = 7), and Red Holstein (n = 16).

Two cows in heat were excluded from the analysis, resulting in a dataset for analysis of 42 cows of the group cubicle housing.

There was no significant difference concerning the lactation number between mountain pasture and cubicle groups (*P* = 0.7). However, DIM was significantly higher in mountain cows in comparison to the cubicle cows (df = 80, *P* < 0.0001)

### Locomotion activity time-budget on mountain pasture

Cows on mountain pasture spent 528.1 min (36.7%) lying, 836.7 min (58.1%) standing and 75.6 min (5.2%) walking during the 24-hour time-budget. During the daytime, the cows spent 6.9 min/h (11.5%) lying, 47.5 min/h (79.2%) standing and 5.6 min/h (9.3%) walking.

### Characteristics of locomotion on mountain pastures in comparison to modern cubicle housing systems

#### 24-hours

Locomotion data for both mountain and cubicle housing groups were compared (Table 2). The cows from the mountain pasture group spent less time lying down (528.1±109.5 min/day vs. 693.3±73.8 min/day; *P*<0.0001) and more walking time (75.6±25.9 min/day vs. 38.8±15.8 min/day; *P* <0.0001), than cubicle cows. Lying bout duration was longer in the cubicle group than in the mountain group (90.9± 15.2 min/bout vs. 74.2 ± 21.1 min/bout; *P* = 0.0001). We observed no significant difference in the number of lying bouts between both groups (*P* = 0.2). However, the number of walking bouts was higher in the mountain group than in the cubicle group (199.1 ± 49.1 vs. 123.8 ± 43.8 bouts per day; *P* < 0.001). Likewise, the number of strides during 24-hour period was higher in the mountain group than in the cubicle group (2040.5 ± 825.3 vs. 916.7 ± 408.6; *P* < 0.001).

**Table 2:** Comparison of RumiWatch® accelerometer variables of mountain pasture and cubicle housing cows during 24-hour locomotion activity budget.

Variable	24-hours								
	Mountain pasture (n = 38)				Cubicle (n = 44)				*P*-value
	Mean	^1^SD	Median	^2^IQR	Mean	SD	Median	IQR	
Lying time, min/d	528.08	109.46	528.08	124.67	693.25	73.75	686.87	122.13	<0.0001
Walking time, min/d	75.56	25.86	71.04	36.69	38.84	15.81	35.67	11.98	<0.0001
Lying bouts, 1/d	7.54	2.29	7	2.38	7.81	1.41	7.75	2	0.211
Walking bouts, 1/d	199.14	49.13	195.25	61.75	123.75	43.75	116.00	31.88	<0.0001
Lying bout duration, min/bout	74.22	21.11	72.35	25.91	90.94	15.19	90.66	18.43	0.0001
Strides, 1/d	2040.50	825.26	1930	1069.25	916.71	408.64	833.50	292.38	<0.0001
Stride frequency, 1/walking bout	26.50	2.01	26.32	2.57	23.37	1.24	23.31	1.51	<0.0001
Stride duration, s/stride	1.77	0.13	1.75	0.15	1.95	0.15	1.93	0.21	<0.0001
Stride distance, m/stride	126.34	18.14	125.54	28.84	142.13	17.79	139.71	21.42	0.0002

^1^ SD standard deviation

^2^ IQR Interquartile range

Cows of the mountain pasture group had shorter stride duration (*P* < 0.0001) and shorter strides (*P* = 0.0002) than cubicle housed cows (177 ± 0.1 s/stride vs 2 ± 0.2 s/stride and 126.3 ± 18.1 vs 142.1 ± 17.8 m/stride, respectively).

Using multivariable linear mixed effect models considering DIM and lactation group in addition to the study group (mountain versus cubicle housing system), number of strides, walking time, walking bouts and stride distance were found to be significantly related to the lactation group (Table 3).

**Table 3:** Coefficient of the variables study group, days in milk and lactation group on the outcome of accelerometer variables during 24-hour locomotion activity budget using multivariable linear mixed effect regression model.

Outcome Variable	Coefficient	SD of the coefficient	*p*-value
**Lying time**			
Study group^1^	163.93	34.37	0.001
Lactation group (2) ^2^	0.08	26.48	0.997
Lactation group (3)	-12.27	26.39	0.643
**Walking time** *			
Study group	-0.71	0.16	0.001
Lactation group (2)	-0.17	0.07	0.013
Lactation group (3)	-0.26	0.07	<0.0001
**Lying bouts** *			
Study group	0.03	0.08	0.711
**Walking bouts** *			
Study group	-0.52	0.13	0.002
Lactation group (2)	-0.10	0.07	0.131
Lactation group (3)	-0.18	0.07	0.007
**Lying bout duration**			
Study group	19.88	6.84	0.015
Lactation group (2)	-8.75	5.30	0.103
Lactation group (3)	-3.98	5.28	0.454
**Strides** *			
Study group	-0.84	0.18	0.001
Lactation group (2)	-0.18	0.07	0.014
Lactation group (3)	-0.29	0.07	<0.0001
**Stride duration**			
Study group	200.02	59.28	0.006
Lactation group (2)	-23.31	35.93	0.519
Lactation group (3)	43.44	35.89	0.230
**Stride distance**			
Study group	14.70	5.96	0.033
Lactation group (2)	12.00	5.26	0.026
Lactation group (3)	1.38	5.23	0.792
**Stride frequency** *			
Study group	-0.13	0.03	0.001

SD = standard deviation

^1^Mountain pasture group as reference level

^2^Lactation group (1) as reference level

*log transformed outcome variables

#### Interval between milking times (daytime)

Table 4 shows the summary statistics of locomotion behavior between the milking times for both groups. Analysis of the daytime activity shows similar results as the 24-hour locomotion activity budget. However, the differences between study groups were larger by comparing the interval between milking times to the 24-hour locomotion activity budget. In addition to this, the number of lying bouts per hour were significantly lower in the pasture group than the cubicle group when considering daytime hours only (0.2±0.1 vs 0.3±0.1; *P* < 0.0001).

**Table 4:** Comparison of RumiWatch® accelerometer variables of mountain pasture and cubicle housing cows during the interval between milking times.

Variable	Interval between milking time								
	Mountain pasture (n = 38)				Cubicle (n = 44)				*P*-value
	Mean	^1^SD	Median	^2^IQR	Mean	SD	Median	IQR	
Lying time, min/h	6.88	3.44	6.49	4.09	21.90	6.77	23.14	9.19	<0.0001
Walking time, min/h	5.61	1.90	5.39	2.31	2.01	1.23	1.74	0.88	<0.0001
Lying bouts, 1/h	0.17	0.09	0.15	0.11	0.26	0.08	0.25	0.10	<0.0001
Walking bouts, 1/h	14.91	3.64	14.75	3.96	6.48	3.51	5.73	2.78	<0.0001
Lying bout duration, min/bout	43.88	17.75	41.30	17.31	87.77	29.10	86.51	29.34	<0.0001
Strides, 1/h	151.15	60.74	142.55	72.88	47.55	31.73	41.05	21.10	<0.0001
Stride frequency, 1/walking bout	26.44	1.98	26.30	2.46	23.31	1.28	23.08	2.42	<0.0001
Stride duration, s/stride	1.77	0.13	1.77	0.15	1.94	0.16	1.91	0.23	<0.0001
Stride distance, m/stride	126.36	18.08	125.40	31.45	142.89	17.69	140.75	17.98	<0.0001

^1^ SD standard deviation

^2^ IQR Interquartile range

## Discussion

According to the authors’ best knowledge, this is the first description of locomotion activity time-budget for dairy cattle housed on mountain pastures. This housing was historically used for small herds of local dual-purpose breeds (for cheese production and heifer rearing), well adapted to mountainous environments. These herds would be housed in closed barns located in the valley during the winter and moved to high pastures in the summer [24]. The most important reasons for mountain summering in this system are the expansion of the forage base, improving the cattle welfare and reducing the farmers’ workload [25]. For dairy cows on mountain pasture, nutritional requirements and climatic conditions are of major welfare concern [26].

A mountain pasture is an ancient traditional housing system and allows cattle to express their normal behavior. Searching for fresh forage is normally associated with the natural behavior of grazing [27]. The data of our study show that the majority of time, cows of the mountain pasture housing group spent 58% of their time budget standing during the 24-hour period, and this increased (79%) during daylight with a higher walking time. Lying, standing and walking times in mountain pasture were significantly different to the cows housed in cubicle systems. On mountain pastures, cows have to walk for long distances when searching for fresh grass, while in cubicle housed cows, the feeding is supplied ad libitum in very close distance to the lying area.

We measured animal activity levels using a valid technology, which is not labor intensive to record the locomotion activity with no severe bias of data interpretation and minimal interference with animal behaviors [23]. Data analysis was performed using averaged 24 hour summaries of two days, which is sufficient to estimate lying behavior accuracy in cattle [28]. Limitations of our study were that external impacts such as sudden exposure to noise caused by a helicopter was not recorded and the potential short period of data collection (48-hour). No sample size calculation was performed, and the number of cows included in the study was based on the number of RumiWatch devices available. We were, however, able to demonstrate highly significant differences for eight of the nine outcome variables investigated, which provides evidence for a large enough sample size.

A further limitation was that data could only be collected during two different seasons. Data of the mountain pasture group were collected in summer (June to August) and of the cubicle housing group in winter (October to March). Some of the observed variation may be explained by this seasonal difference of data acquisition. In the cubicle housing group, no access to pasture was permitted to the cows from at least 3 d before until the end of data collection. It cannot be fully excluded that this sudden loss of outdoor access may have led to an initial period of frustration for these animals that could have influenced their locomotor behavior.

Lying is one of the most important behavioral parameters of dairy cows and can provide insights into cow welfare and as an indicator of cow and stall comfort [29, 30]. A previous study showed that pastures provide an adequately comfortable lying area [31]. Inadequate lying and prolonged standing time increase the likelihood of lameness developing in cattle. Galindo and Broom [32] found that when cows spent >45% of the day standing, it was related to a higher incidence of lameness. More recent study reported that longer standing time and longer standing bouts after calving were associated with increased odds of developing sole hemorrhages and sole ulcers in free-stall housed dairy cows [33]. However, our data suggested that mountain pasture based cows spent more time standing than cubical housed cows. No obvious signs of lameness were observed in the mountain pasture based cows over the course of the study, however, further researcher regarding the long term foot health of cows kept in such systems is needed. It is likely that this increase in standing behavior is due to mountain pasture based cows expressing normal behaviors that are not observed in cubical housed systems, like searching for fresh forage. Mountain pasture based cows may be spared the foot health issues associated with increased standing as seen in free-stall housing due to the more natural flooring type.

All activity variables measured by RumiWatch® accelerometers were comparable to the previous study of freestall housing systems. Mean daily lying time (693.3 min/d), number of the lying bouts (7.8 bouts/d) and walking time (38.8 min/d) time supports values of previous studies that recorded the data on healthy dairy cows kept on free-stall housing [28, 34]. However, the duration of the lying bouts (90.9 min/bouts) observed in this study was longer than that reported by Beer et al. [34] and similar to Ito et al. [28]. The extended variables of number of strides (916.7 1/d), stride distance (142.1 m/stride) and stride duration (2 s/stride) were comparable to the previous study which observed healthy dairy cows in the cubicle housing system [34]. The shorter strides observed on mountain pasture may be explained by the rough uneven terrain. Walking time and number of strides were almost triple in mountain pastures as compared to cubicle housing systems. First lactation dairy cows showed higher walking time as compared to cows of second or lactation or higher. Adult cows tended to lie longer than first lactation cows kept in cubicles [35].

## Conclusions

The results of this study show that the mountain pasture housed cows spent 58% of time during the 24-hour period standing and this increased even to 79% during daytime. Mountain pastured cows had a higher locomotor activity level in comparison to the cubicle housed cows. Lying markedly less than 12 hours per day seems to represent the normal behavior of pastured cows searching for fresh grass based on 48 hours measurements.

## Supporting information

S1 Dataset Animals, measured data and statistical analysis Excel data. Data used for statistics.
(XLSX)
